# Hypoxia-inducible lipid droplet-associated protein inhibits adipose triglyceride lipase

**DOI:** 10.1194/jlr.M082388

**Published:** 2018-01-11

**Authors:** Krishna M. Padmanabha Das, Lisa Wechselberger, Márton Liziczai, Montserrat De la Rosa Rodriguez, Gernot F. Grabner, Christoph Heier, Roland Viertlmayr, Claudia Radler, Jörg Lichtenegger, Robert Zimmermann, Jan Willem Borst, Rudolf Zechner, Sander Kersten, Monika Oberer

**Affiliations:** Institute of Molecular Biosciences,* University of Graz, 8010 Graz, Austria; Division of Human Nutrition† Wageningen University, Wageningen, The Netherlands; Laboratory of Biochemistry and Microspectroscopy Research Facility,** Wageningen University, Wageningen, The Netherlands; BioTechMed-Graz,§ 8010 Graz, Austria

**Keywords:** hypoxia-inducible gene-2, intracellular lipolysis, adipocytes, lipolysis and fatty acid metabolism, triglycerides

## Abstract

Elaborate control mechanisms of intracellular triacylglycerol (TAG) breakdown are critically involved in the maintenance of energy homeostasis. Hypoxia-inducible lipid droplet-associated protein (HILPDA)/hypoxia-inducible gene-2 (Hig-2) has been shown to affect intracellular TAG levels, yet, the underlying molecular mechanisms are unclear. Here, we show that HILPDA inhibits adipose triglyceride lipase (ATGL), the enzyme catalyzing the first step of intracellular TAG hydrolysis. HILPDA shares structural similarity with G0/G1 switch gene 2 (G0S2), an established inhibitor of ATGL. HILPDA inhibits ATGL activity in a dose-dependent manner with an IC_50_ value of ∼2 μM. ATGL inhibition depends on the direct physical interaction of both proteins and involves the N-terminal hydrophobic region of HILPDA and the N-terminal patatin domain-containing segment of ATGL. Finally, confocal microscopy combined with Förster resonance energy transfer-fluorescence lifetime imaging microscopy analysis indicated that HILPDA and ATGL colocalize and physically interact intracellularly. These findings provide a rational biochemical explanation for the tissue-specific increased TAG accumulation in HILPDA-overexpressing transgenic mouse models.

In times of nutrient surplus, mammals store triacylglycerols (TAGs) in lipid droplets (LDs) of white adipose tissue. Under starving conditions, a process termed intracellular lipolysis hydrolyzes TAG stores leading to the release of fatty acids and glycerol, which are utilized in other tissues for energy production as well as lipid and membrane biosynthesis. Dysregulation of the coordinated processes of TAG synthesis and mobilization leads to various pathologies, including metabolic syndrome, atherosclerosis, (nonalcoholic) fatty liver disease, certain forms of cancer (including cervical and liver cancer), and cancer-associated cachexia (1, 2).

Lipolysis involves the consecutive action of three major lipases called adipose triglyceride lipase (ATGL), hormone-sensitive lipase (HSL), and monoacylglycerol lipase (MGL). The activity of ATGL, which hydrolyzes TAGs into diacylglycerols and unesterified fatty acids, is modulated by numerous transcriptional and posttransciptional regulatory processes, including interaction with protein coregulators (3). The coactivator protein, comparative gene identification-58 (CGI-58) [also termed α-beta hydrolase domain-containing 5 (ABHD5)], activates, whereas G0S2, the gene product of the G0/G1 switch gene 2, inhibits the TAG-hydrolyzing activity (4, 5). In humans and mice, the ATGL inhibitor, G0S2, is a 103 amino acid protein with high expression levels in adipose tissues, bone marrow, skeletal muscle, and liver (5–8). Direct protein-protein interaction of G0S2 with the patatin domain within ATGL mediates the selective inhibition of enzyme activity (9, 10). G0S2 is a PPARγ target gene (8) and, additionally, highly upregulated during hypoxia (11). Tissue-specific overexpression or deletion of G0S2 in mice strongly affects TAG accumulation in various tissues (12).

Recent work identified another LD-associated protein that is structurally related to and similarly regulated as G0S2 named hypoxia-inducible LD-associated protein (HILPDA) (13–16). HILPDA comprises a 63 amino acid protein and is also known as hypoxia-inducible gene-2 (Hig-2). It was originally identified in cervical cancer cells upon hypoxic stress (17). Similar to G0S2, HILPDA expression is regulated by hypoxia-inducible factor-1, PPARs, and β-adrenergic stimulation (13, 15, 18). The highest expression levels are observed in brain, endocrine tissues, muscle (skeletal muscle and heart), lung, liver, and adipose tissue (13, 19, 20). Additionally, HILPDA is abundantly expressed in many cancer tissues and atherosclerotic plaques (13, 20).

The physiological function of HILPDA remains unclear. Its regulation by PPARs and localization on LDs suggested a role in neutral lipid metabolism (18). Consistent with this hypothesis, *Hilpda* gene deletion in mice resulted in loss of LD formation in macrophages, reduced fat pad weight, and lower hepatic lipid levels, while overexpression caused a steatotic phenotype in hepatocytes (13, 14, 16, 18). Whether HILPDA directly interferes with the enzymatic catabolism of TAGs remains controversial (10, 11). Considering the structural similarities between HILPDA and G0S2, we tested to determine whether HILPDA acts as an inhibitor of ATGL enzyme activity. We show that HILPDA directly binds ATGL and inhibits its TAG hydrolase (TGH) activity in vitro with an IC_50_ value in the low micromolar range. The interaction of both proteins involves the patatin domain-containing region of ATGL and the N-terminal predominantly hydrophobic region of HILPDA. Furthermore, we show a physical interaction between HILPDA and ATGL by Förster resonance energy transfer (FRET)-fluorescence lifetime imaging microscopy (FLIM) analysis.

## MATERIALS AND METHODS

### Materials

If not stated otherwise, chemicals, antibiotics, and buffers were obtained from Sigma-Aldrich (St. Louis, MO) or Carl Roth GmbH (Karlsruhe, Germany); columns for protein purification were from GE Healthcare Life Sciences (Chicago, IL). The [9,10-^3^H] triolein was obtained from PerkinElmer Life Sciences (Waltham, MA). Triolein, phosphatidylcholine, phosphatidylinositol, 1(rac)-oleoylglycerol, oleoyl-CoA, and free glycerol detection reagents were purchased from Sigma-Aldrich.

### Sequence analysis

Amino acid sequences for HILPDA and G0S2 were retrieved from the UniProt knowledgebase (21), the sequence alignment and phylogram were generated with Clustal Omega (22). Structure prediction was carried out in Phyre2 (23), the figure displaying the predicted structure was generated using PyMOL (24).

### Cloning of HILPDA, ATGL, and HSL constructs

All biochemical experiments were performed with human HILPDA (hHILPDA). Fluorescence microscopy and FRET-FLIM were carried out with mouse HILPDA (mHILPDA). The cDNA clone for hHILPDA was obtained from Dharmacon (accession, BC112183.1; MGC, 138388). The expression cassette was cloned into a modified pSUMO vector (9) with a 6XHis tag and a TEV cleavage site, following the Gibson assembly cloning protocol (25) (New England BioLabs, Ipswich, MA) using primers mentioned in **Table 1**. N-terminal truncations were generated following the Gibson assembly protocol, and C-terminal truncations were made using the Q5® site-directed mutagenesis kit (New England BioLabs). The coding sequence of mouse HSL (mHSL) was amplified by PCR from the pcDNA4/HisMaxA-mHSL construct described in (4) using the primers listed in Table 1. The PCR product was cloned into pECFP-N1 vector (Clontech, Mountain View, CA) using the *Xho*I and *Sac*II restriction sites. The coding sequence for mouse ATGL (mATGL), encoding residues 1-288, and superfolder (sf)-GFP was amplified using primers described in Table 1. The resulting DNA fragments were cloned into the pST50 vector (26) following the Gibson assembly cloning protocol. The correctness of all generated inserts was verified by DNA sequencing.

**TABLE 1.** Primers used for cloning of HILPDA variants, HSL and ATGL

Construct	Forward Primer (5′-3′)	Reverse Primer (5′-3′)	Vector
hHILPDA_1-63	GTATTTTCAGGGCGCCATGGCCATGAAGCATGT­GTTGAACC	AGTGGTGGTGGTGGTGGTG­CCAACGGTGCT­CAG­CTTGTC	pSumo
hHILPDA_1-20	CTCCATCTTCTAGAGAGTGATGTAGTCCC	AGTAGGGTCAGTACCACAC	pSumo
hHILPDA_1-24	TAGAGTGATGTGATCCCTAGAGGGCTTAC	ACGAAGATGGAGAGTAGG	pSumo
hHILPDA_1-28	GTCCCTAGAGTGATTACTAGAGAG	TCCATCACTCTAACGAAG	pSumo
hHILPDA_20-63	GTATTTTCAGGGCGCCATGGGCTTCGTTA­GAGTGATGG	AGTGGTGGTGGTGGTGGTGCTCACATGCTT­CTG­GATGG	pSumo
hHILPDA_25-63	GAGTCCCTAGAGGGCTTACTAGAG	GCCCATGGCGCCCTGAAA	pSumo
hHILPDA 1-63	GCATGGACGAGCTGTACAAGGAAAACCTGT­ATTTT­CAGGG	GGCTGATTATGATCAGTTATTCAGTGATGG­TGAT­GGTGATGCATGCTTCTGGATGGATG	pGFPC1
mHSL	GTCACTCGAGGCCACCATGGATTTACGCACGATGAC	GACTCCGCGGGTTCAGTGGTGCAGCAGGCG	pECFP-N1
mATGL_1-288	AGGTGGCGGAGAAAACCTGTATTTTCAGGGCATG	GCGGGTGGCTCCAAGCGCTGCCGGCATCTT­CTT­CGCCGGCACG	pST50
mHILPDA	GCCTCGAGACCATGAAGTTCATGCTGAAC	ATGGTACCCTGCACTCCTCGGGATGG	pEGFP-N2
mATGL	GCCTCGAGACCATGTTCCCGAGGGAGACCAAG	ATGGTACCGCAAGGCGGGAGGCCAGG	pEGFP-N2
mCherry	ATGGTACCATGGTGAGCAAGGGCGAG	AGCGGCCGCTCACTTGTACAGCTCGTC	pEGFP-N2
sYFP2	ATGGTACCATGGTGAGCAAGGGCGAG	AGCGGCCGCTTACTTGTACAGCTCGTC	pEGFP-N2
mTurquoise2	ATGGTACCATGGTGAGCAAGGGCGAG	AGCGGCCGCTCACTTGTACAGCTCGTC	pEGFP-N2

### Bacterial expression and purification of hHILPDA and mATGL

The expression plasmids coding for 6XHis-smt3-TEV-hHILPDA or truncated variants were transformed into BL21-CodonPlus (DE3)-RIL competent cells (Stratagene, La Jolla, CA), and the cultures were grown at 37°C in Luria-Bertani medium containing kanamycin (50 μg/ml) to an OD_600_ of 0.6. Cells were cooled down to 16°C and induced with 0.5 mM isopropyl β-D-1-thiogalactopyranoside. Following overnight induction, cells were harvested and lysed in lysis buffer [20 mM Tris/HCl, 300 mM NaCl, and 0.05% IGEPAL® CA-630 (pH 7.8)] by sonicating at 50% amplitude for 5 min (SONOPLUS HD2070; Bandelin, Berlin, Germany). The soluble fraction was collected after centrifugation at 40,000 *g* for 40 min. The cleared lysate was loaded to a preequilibrated HisTrap FF column, washed with 10 column volumes of wash buffer [20 mM Tris, 300 mM NaCl, 30 mM imidazole, and 10% glycerol (pH 7.8)] and eluted over a linear gradient against the same buffer, yet with 300 mM imidazole. Fractions containing the purified fusion protein were concentrated and subjected to gel filtration chromatography on a HiLoad 26/60 Superdex 200 preparatory column using SEC buffer [15 mM Na_2_HPO_4_, 5 mM KH_2_PO_4_, 300 mM NaCl, 1 mM EDTA, and 1 mM DTT (pH 6.8)]. The peak corresponding to the fusion protein was concentrated, quantified, and used for TGH assay.

mATGL288 used for TGH assay and sf-GFP-mATGL288 used for microscale thermophoresis (MST) measurements were expressed in ArcticExpress (DE3) competent cells (Agilent Technologies, Palo Alto, CA) for 24 h at 10°C. To purify the sf-GFP-mATGL_288 variant, the bacterial pellet was resuspended in ATGL lysis buffer [80 mM K_2_HPO_4_, 20 mM KH_2_PO_4_, 100 mM KCl, 10% glycerol, 1 mM TCEP, 10 mM ATP, 10 mM MgCl_2_, and 1 mg/ml n-dodecylphosphocholine (pH 7.5)], lysed by sonication, and centrifuged. The supernatant was loaded on to a preequilibrated Strep Trap HP column and washed with ATGL wash buffer [250 mM Tris/HCl (pH 7.5), 500 mM KCl, 10% glycerol, 250 mM sucrose, 1 mM TCEP, 10 mM MgCl_2_, and 10 mM ATP]. The bound protein was eluted using ATGL elution buffer [100 mM Tris (pH 7.5), 50 mM KCl, 10% glycerol, 250 mM sucrose, 1 mM TCEP, 1 mM EDTA, and 10 mM desthiobiotin]. The eluted pro­tein was further purified using anion exchange chromatography over a ResourceQ column using the same buffer as above in a 0–500 mM KCl gradient. The peak fractions containing the ATGL variants, as judged by SDS-PAGE, were concentrated and loaded onto a Superdex 200 Increase 10/300 GL gel filtration column using ATGL SEC buffer [100 mM Tris/HCl (pH 7.5), 100 mM KCl, 10% glycerol, 1 mM TCEP, 1 mM EDTA, 1 mg/ml n-dodecyl­phosphocholine, and 100 mM sucrose]. The ATGL-containing fractions were quantified and further used for MST experiments. Expression and purification of hG0S2 and mCGI-58 were done following previously described protocols (9, 10).

### Expression of recombinant proteins in HEK293 cells and preparation of cell extracts for coimmunoprecipitation and enzyme activity assays

HEK293T cells were cultivated in DMEM (GIBCO, Invitrogen Corp., Carlsbad, CA) containing 10% FCS (Sigma-Aldrich) at standard conditions (37°C, 5% CO_2_, 95% humidified atmosphere). Cells were transfected with 6 μg DNA (or 3 μg each in cotransfection experiments) complexed to Metafectene (Biontex GmbH, Munich, Germany) in serum-free DMEM. After 4 h, the medium was replaced by DMEM supplemented with 10% FCS. For coimmunoprecipitation experiments, cells were washed three times with PBS, collected using a cell scraper, harvested by brief centrifugation, washed twice with PBS, and disrupted in lysis buffer [50 mM Tris/HCl (pH 7.4), 150 mM NaCl, 1 mM EDTA, and 1% NP40]. Cells were vortexed, incubated on ice for 30 min, and pressed five times through a 23 gauge needle using a 1 ml syringe for mechanical lysis. Lysed cells were centrifuged for 15 min at 20,000 *g* at 4°C and the supernatants were used for immunoprecipitation experiments. Protein samples for enzyme activity assays were prepared by sonication of transfected cells in 0.25 M sucrose, 1 mM EDTA, and 1 mM DTT supplemented with 20 μg/ml leupeptin, 2 μg/ml antipain, and 1 μg/ml pepstatin. Perinuclear supernatants were prepared by centrifugation at 1,000 *g* for 10 min and were used for enzyme activity assays.

### Determination of protein concentration

Protein concentration of cell lysates and purified proteins was determined using the Bio-Rad protein assay kit (Bio-Rad Laboratories Inc., Hercules, CA) according to the manufacturer’s instructions. BSA (Thermo Fisher Scientific, Waltham, MA) was used for the generation of standard curves. Concentrations of some purified proteins were additionally obtained by the absorbance at 280 nm using the NanoDrop® ND-1000 spectrophotometer (PEQLAB Biotechnologie GmbH, Erlangen, Germany).

### Immunoblotting

Fifty micrograms of each bacterial lysate containing hHILPDA and truncated variants or ATGL lysates were loaded and run on a denaturing 12% SDS-PAGE gel and transferred to nitrocellulose membrane. Immunoblot was performed using mouse Penta His antibody (34660; Qiagen, Dusseldorf, Germany) or StrepMAB-Classic mouse monoclonal antibody (2-1507-001; IBA Lifesciences, Goettingen, Germany) followed by ECL anti-mouse IgG secondary antibody (GE Healthcare Life Sciences). The blot was developed using Amersham ECL Western blotting detection reagent (GE Healthcare Life Sciences).

In case of coimmunoprecipitation, immunoblot was performed using either monoclonal anti-Flag® M2-peroxidase HRP antibody (Sigma-Aldrich) or Penta His antibody (Qiagen) followed by mouse TruBlot® anti-mouse Ig HRP secondary antibody (Rockland, Limerick, PA).

### TGH assays and IC_50_ determination

TGH assays were performed essentially as described (27), yet on a smaller scale. In order to prepare bacterial lysates, harvested pellets were resuspended in sucrose solution [250 mM sucrose, 1 mM EDTA, 1 mM DTT, 20 μg/ml leupeptin, 2 μg/ml antipain, and 1 μg/ml pepstatin (pH 7.0)] and disrupted by sonication as described earlier (9, 28, 29). Five micrograms of purified HILPDA protein, 25 μg total protein of ATGL cell lysate, and 1 μg of purified mCGI-58 were incubated with [^3^H]triolein-containing substrate mix. Five micrograms of purified hG0S2, instead of purified HILPDA, were used in positive controls. To assess TGH activity of mHSL, the lipase was expressed as fusion protein (HSL-ECFP) in HEK293 cells and perinuclear supernatants were prepared as an enzyme source, as described above. Twenty-five micrograms total protein of mHSL cell lysate were incubated in the absence and presence of purified HILPDA. LPL was purified from bovine milk, essentially as described (30), and 33 ng of LPL protein were used for the assay. In order to prepare the TAG substrate, 1.67 mM triolein, 10 μCi/ml [9,10-^3^H]triolein, and 190 μM of phosphatidylcholine/phosphatidylinositol in a ratio of 3:1 were sonicated and the concentration was adjusted with the buffer. Twenty-five microliters of the respective protein preparation were incubated with 25 μl of [^3^H]triolein substrate at 37°C for 1 h. The reaction was terminated by adding 650 μl of methanol/chloroform/heptane (10:9:7) and 200 μl of 100 mM potassium carbonate buffer, pH 10.5. After extraction and centrifugation, the radioactivity in 200 μl of the upper phase was determined by liquid scintillation counting. Dose response experiments of stimulated mATGL288 activity using increasing concentrations of hHILPDA (44 nM to 44 μM) were carried out to determine the the IC_50_. Curve fitting to the data and calculation of the IC_50_ value were implemented using GraphPad Prism 5 (GraphPad Software Inc.). The activities derived from TGH assays (measured from triplicates and at least two independent biological replicates) are represented as mean ± SD. Statistical significance was tested by the Student’s unpaired two-tailed *t*-test. Data are considered to be significantly different for *P* < 0.05 (*), *P* < 0.01 (**), and *P* < 0.001 (***).

### Monoacylglycerol hydrolase activity assay

hHILPDA was also analyzed for its ability to inhibit monoacyl­glycerol hydrolase activity of human MGL (hMGL) by following the assay protocol described earlier (31) using rac-1-3 oleoyl­glycerol as the substrate. A concentration of 5 nM of hMGL and hHILPDA concentrations ranging from 2.5 nM to 250 nM were tested for the inhibition of hMGL.

### Coimmunoprecipitation

Flag agarose beads were prepared by washing with lysis buffer [50 mM Tris-HCl (pH 7.4), 150 mM NaCl, 1 mM EDTA, and 1% NP40] four times. Three hundred and fifty micrograms of HEK293 cell lysate were incubated with 10 μl of anti-Flag agarose beads (ANTI-FLAG® M2 affinity gel; Sigma-Aldrich) overnight at 4°C top in a sample shaker. Beads were washed four times with lysis buffer, and the beads were boiled in 30 μl of 2× SDS sample buffer [125 mM Tris-HCl (pH 6.8), 5% SDS, 0.004% bromophenol blue, 10% β-mercaptoethanol, and 20% glycerol]. Fifteen microliters of these samples were loaded onto 12% SDS-PAGE gel and subjected to immunoblotting, as detailed earlier.

### MST

Protein-protein interaction studies using MST were carried out on a Monolith NT.115 instrument (Nanotemper, Munich, Germany). Increasing concentrations (1.1 μM to 487 μM) of purified smt3-tagged hHILPDA in buffer [20 mM phosphate buffer, 300 mM NaCl, 1 mM EDTA, and 1 mM DTT (pH 7.0)] were titrated against constant amounts of sf-GFP-mATGL_288 (0.01 μM). Samples were loaded into premium capillaries and measurements were performed in triplicate at 20% LED power and 40% MST power at room temperature. A constant concentration of 1% BSA was maintained in all the samples to avoid nonspecific interactions. Data analysis was performed with Monolith software (Nanotemper) using both thermophoresis and T jump parameters.

### Cloning of mHILPDA and mATGL for fluorescence microscopy

Plasmids of mHILPDA_sYFP2, mHILPDA_mCherry, and mATGL_mTurquoise2 were constructed by fusing the full-length cDNA into pEGFP-N2 (Clontech) and substituting the EGFP sequence by the sequence of the fluorescent proteins, mCherry, sYFP2, or mTurquoise2. Briefly, RNA from mouse white adipose tissue and liver was reverse transcribed with a First Strand cDNA synthesis kit (Thermo Fisher Scientific) and amplified with Phusion high-fidelity DNA polymerase (Thermo Fisher Scientific) using the primers mentioned in Table 1. The PCR products were cloned into pEGFP-N2 vector using the *Xho*I and *Kpn*I-HF (New England Biolabs) restriction sites. Afterwards, MAX Efficiency® DH5α™ competent cells (Invitrogen) were transformed by heat-shock and grown in Luria-Bertani agar plates with kanamycin. The vector was isolated using Qiagen plasmid maxi kit (Qiagen) according to the manufacturer’s instructions. The EGFP fragment was then excised from the pEGFP-N2 parent vector by enzyme digestion with *Kpn*I-HF and *Not*I-HF. The vector was gel-purified with QIAquick gel extraction kit (Qiagen) and the fragments of mCherry, sYFP2, or mTurquoise2 (32) were ligated using T4 DNA ligase (Thermo Fisher Scientific) into the pEGFP-N2 vector, which was digested with *Kpn*I and *Not*I. The resulting constructs used were mHILPDA fused to either mCherry or sYFP2 and mTurquoise2 fused to mATGL.

### Visualization of mHILPDA and mATGL colocalization and interaction

Hepa 1-6 cells were cultured in DMEM (Lonza, Verviers, Belgium) supplemented with 10% fetal calf serum (Lonza) and 1% penicillin/streptomycin (Lonza) at standard conditions (37°C, 5% CO_2_, 95% humidified atmosphere). Cells were seeded on a collagen-coated 8-well removable chamber (Ibidi, Martinsried, Germany) and grown for 24 h before transfection. Transfections were performed with 700 ng of single or mixed plasmid DNA complexed to polyethylenimine (Polyscience Inc., Warrington, PA) in serum-free DMEM. After 5 h, the medium was replaced by serum-free DMEM supplemented with 1.5% fatty acid-free BSA (Roche Applied Sciences) and 1.2 mM of oleic acid and palmitic acid mix in a 2:1 oleic acid:palmitic acid ratio for 24 h to promote LD formation. Cells were washed with PBS, fixed for 20 min with 4% formaldehyde and mounted with Vectashield-H (Vector Laboratories). For colocalization analysis, Hepa 1-6 cells were transfected with mATGL_mTurquoise2 and mHILPDA_mCherry. For FRET-FLIM analysis, Hepa 1-6 cells were transfected with mATGL_mTurquoise2 and mHILPDA_sYFP2 plasmids.

Colocalization imaging was performed on a Leica TCS SP5 X system equipped with a 63× 1.20 NA water-immersion objective lens. Images were acquired sequentially 1,024 × 1,024 pixel scans with pinhole set at 1 airy unit. mTurquoise2 was excited at 458 nm and fluorescence emission was detected using internal Hybrid (HyD) in a spectral window of 460–500 nm. mCherry was excited at 561 nm and detected using HyD in a spectral window of 588–655 nm. Images were processed using Fiji. Briefly, a Gaussian blur of 1 unit was applied; background was measured from the nucleus and subtracted.

FRET-FLIM was performed on a Leica TCS SP8 X confocal set-up, including a picosecond pulsed diode laser, LDH-D-C-440 (PicoQuant), resulting in excitation pulses of 10 ps at a repetition of 40 MHz. Fluorescence emission was detected using HyD detectors with 100 ps time resolution and collected in a spectral window of 450–495 nm for the donor (mTurquoise2). The acceptor (sYFP2) was excited using a 40 MHz tunable supercontinuum laser at 514 nm and the fluorescence was detected from 540 to 600 nm. The signal output from the HyD detector was coupled to an external time-correlated single photon counting module (Becker & Hickl) for acquiring FLIM data. Typical images had 128 × 128 pixels (pixel size, ±300 nm) and 256 time channels per pixel with an acquisition time of 80 s per image. From the time-resolved fluorescence intensity images, the fluorescence decay curves were calculated for each pixel and fitted with a double-exponential decay model using the SPCImage v6.4 software (Becker & Hickl). Fitting was performed without fixing any parameters. FRET-FLIM analysis provided fluorescence intensity as well as false-colored fluorescence lifetime images. The raw data were subjected to the following criteria to analyze and omit false-positive negatives in the fluorescence lifetime scoring: minimum photon count per pixel of 1,200 photons, two-component analysis, goodness of fit (χ^2^ <2), and fluorescence lifetime range of 500–3,500 ps. For data analysis, we set pixel binning at 1 to have a sufficient number of photons per pixel required for accurate fluorescence lifetime analysis.

## RESULTS

### HILPDA inhibits human and mouse ATGL under basal and CGI-58-stimulated conditions

**Fig. 1.**
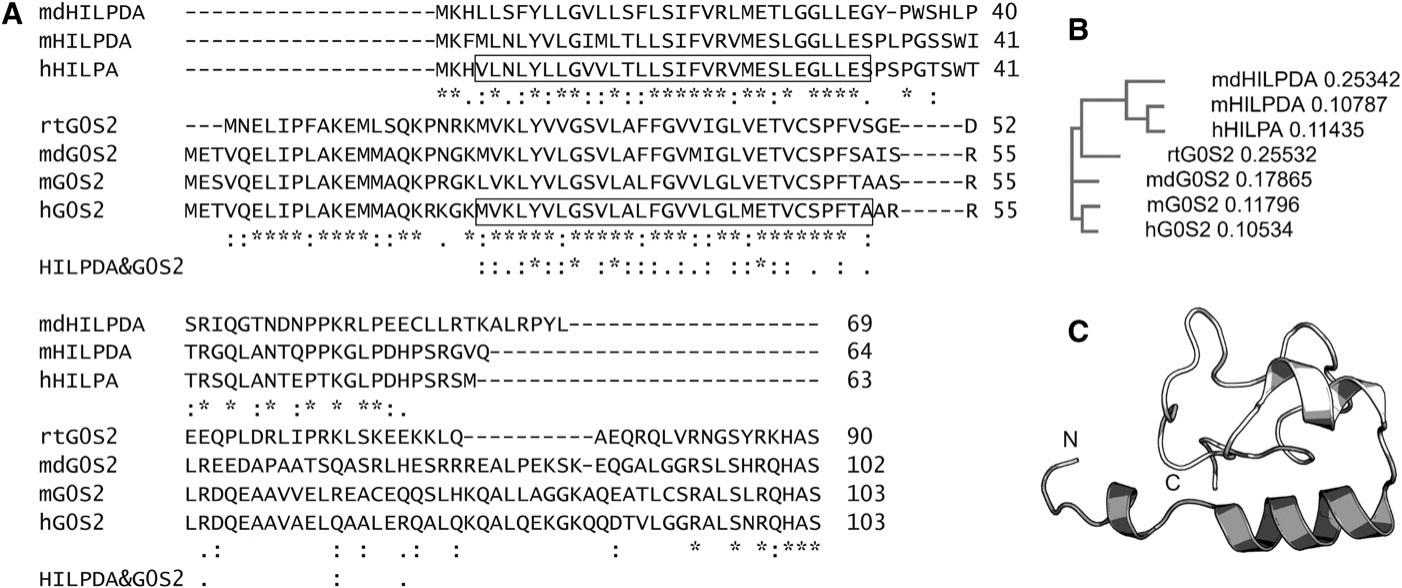
Sequence and structural analysis of HILPDA. A: Sequence alignment of HILPDA and G0S2 from different species (md, *Monodelphis domestica*; m, *Mus musculus*; h, *Homo sapiens*; rt, *Rhincodon typus*). Residues highlighted by a box correspond to highly similar regions in the sequences of hHILPDA and hG0S2. Similarities (“:” highly similar and “.” similar) and identities (*) between HILPDA and G0S2 proteins are indicated directly below the sequences of the two proteins, those of both proteins are indicated in an extra line (bold). B: Phylogram of aligned sequences as in A. C: Secondary and tertiary structure prediction of HILPDA using Phyre2. Residues 2-23 aligned with the integrin αl transmembrane domain (pdb-code 2me3; unpublished observations) and are colored dark gray.

**Fig. 2.**
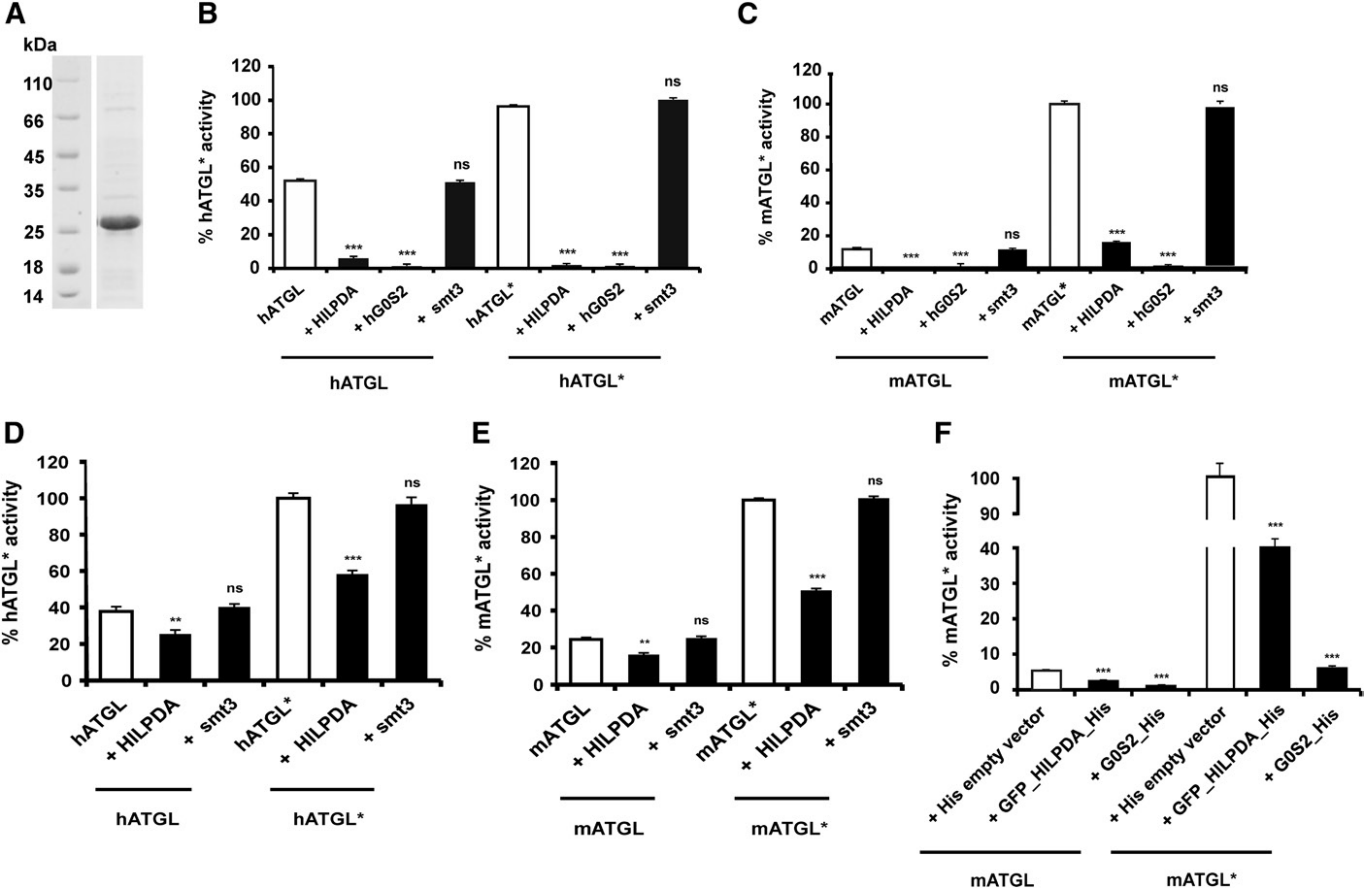
hHILPDA inhibits hATGL and mATGL under basal and CGI-58 stimulated conditions. A: Coommassie stained SDS-PAGE gel of purified smt3-HILPDA fusion protein (22 kDa). B–E: Activity assays were performed in the absence and presence (indicated by an asterisk, i.e., hATGL*) of 1 μg purified CGI-58. Twenty-five micrograms of ATGL lysates and 5 μg of purified HILPDA were used in all assays. Five micrograms of purified hG0S2 or 5 μg of purified smt3 were used wherever it is specified. Values corresponding to 100% ATGL activity upon activation with CGI-58 (i.e., hATGL*) are mentioned in parentheses below. TGH activity of bacterial cell lysates expressing human [343 nmol FA/h*mg protein (B)] or mouse [635 nmol FA/h*mg protein (C)] ATGL288 in the absence or presence of purified CGI-58, HILPDA, or smt3. TGH activity of HEK293 cell lysates expressing human [634 nmol FA/h*mg protein (D)] and mouse [638 nmol FA/h*mg protein (E)] ATGL in the absence and presence of purified CGI-58, HILPDA, or smt3. F: HEK293 cell lysates coexpressing mATGL and HILPDA were tested for TGH activity levels under basal and CGI-58-stimulated conditions. HEK293 cell lysates coexpressing mATGL (395 nmol FA/h*mg protein) and hG0S2 or empty vector served as controls. Statistical significance in comparison with ATGL* (white bar) was assigned according to the scheme: *P < 0.05, **P < 0.01, ***P < 0.001 representing three independent experiments. ns, not significant.

The 103 amino acid protein, G0S2, suppresses TAG catabolism via direct inhibition of the TAG-hydrolyzing activity of ATGL (5). In addition to similarities in expression, regulation, and intracellular localization, structural comparison of HILPDA and G0S2 revealed that the proteins also share amino acid sequence similarities (approximately 22% identity, 40% similarity). Accordingly, web portals for protein structure (23) predicted similar secondary structures and similar spatial distributions of structural elements (**Fig. 1**). To reveal potential functional similarities, we tested to determine whether HILPDA is able to inhibit ATGL in vitro in a similar manner as G0S2. Previous studies identified the patatin domain-containing region of ATGL to be sufficient for catalysis, activation by CGI-58 and inhibition by G0S2 (10). Therefore, we hypothesized, that this region would also present the potential interface for HILPDA and used ATGL variants truncated after residue 288 (hATGL288, mATGL288) in our assays. To test the influence of HILPDA on ATGL, we first incubated *Escherichia coli* cell lysates overexpressing a truncated, yet fully active, variant of hATGL with purified human HILPDA (**Fig. 2A**). Purified smt3 and hG0S2 served as controls. As shown in Fig. 2B, HILPDA as well as G0S2 inhibited the TAG activity of hATGL by ∼90%, while smt3 had no effect on enzyme activity. Similarly to hATGL, HILPDA also inhibited mATGL in the absence or presence of CGI-58 (Fig. 2C). To investigate whether the inhibitory effect of HILPDA can be reproduced when recombinant proteins are expressed in mammalian cells, HEK293 lysates overexpressing hATGL and mATGL were tested for inhibition by HILPDA. Again, HILPDA inhibited both mouse and human ATGL in the presence or absence of CGI-58 (Fig. 2D, E). The overexpression of ATGL in the lysates was verified by immuno­blotting analysis (data not shown). When mATGL was coexpressed with HILPDA in HEK293 cells, mATGL activity was ∼60% lower than in cells expressing only ATGL (Fig. 2F). Again, HILPDA inhibited ATGL activity in the presence or absence of CGI-58. mATGL coexpression with hG0S2 reduced TGH activity by ∼90%, suggesting a more potent ATGL inhibition effect of G0S2 than of HILPDA.

### The N-terminal region of HILPDA is required and sufficient to inhibit ATGL activity

**Fig. 3.**
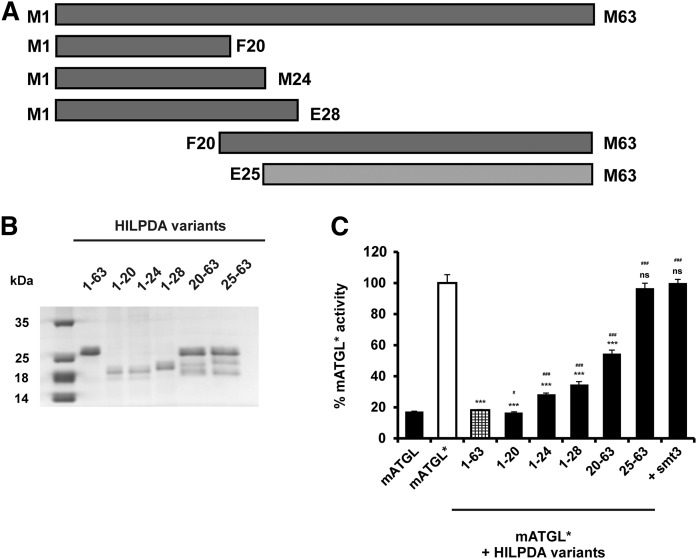
The N-terminal region of HILPDA is essential for ATGL inhibition. A: Graphical representation of full-length and C- and N-terminal truncated versions of HILPDA. Variants capable of ATGL inhibition are colored in dark gray. B: Coommassie stained SDS-PAGE gel of purified HILPDA variants expressed in *E. coli*. C: HILPDA variants were tested for their ability of ATGL inhibition. Activity assays were performed in the presence of CGI-58, as indicated by an asterisk (mATGL*). Five micrograms of purified HILPDA protein was mixed with 25 μg of mATGL288 lysate and 1 μg of purified mCGI-58. Purified smt3 was used as a negative control. One hundred percent ATGL activity corresponds to 635 nmol FA/h*mg protein. Statistical significance in comparison with ATGL* (white bar) was assigned according to the scheme: *P < 0.05, **P < 0.01, ***P < 0.001 and, in comparison with HILPDA_FL (checked bar), according to the following scheme #*P* < 0.05, ##*P* < 0.01, ###*P* < 0.001 representing three independent experiments. ns, not significant.

The N-terminal region of G0S2 has been identified to be essential for inhibition of ATGL (5, 9). This region shares the highest sequence similarity with HILPDA (Fig. 1A). To identify sequence motifs within the HILPDA polypeptide required for ATGL inhibition, we generated several C- and N-terminal truncations of the protein (**Fig. 3A**). All constructs were expressed in *E. coli*, purified, and subjected to an SDS-PAGE gel to estimate yield and purity (Fig. 3B). Bacterial lysates overexpressing mATGL288 were used to test hHILPDA variants for their ability to inhibit the TAG-hydrolyzing capacity of ATGL. C-terminal truncation variants of hHILPDA (M1-E28, M1-M24, and M1-F20) inhibited ATGL to a similar extent as the full-length protein (Fig. 3C). In contrast, the HILPDA peptide, F20-M63, showed a reduced ability to inhibit ATGL, whereas the truncation E25-M63 lost the ability to inhibit ATGL activity (Fig. 3C). These findings suggest that N-terminal segments in the HILPDA polypeptide, which share highest sequence conservation to G0S2, mediate the inhibition of ATGL activity.

### HILPDA directly binds to ATGL

**Fig. 4.**
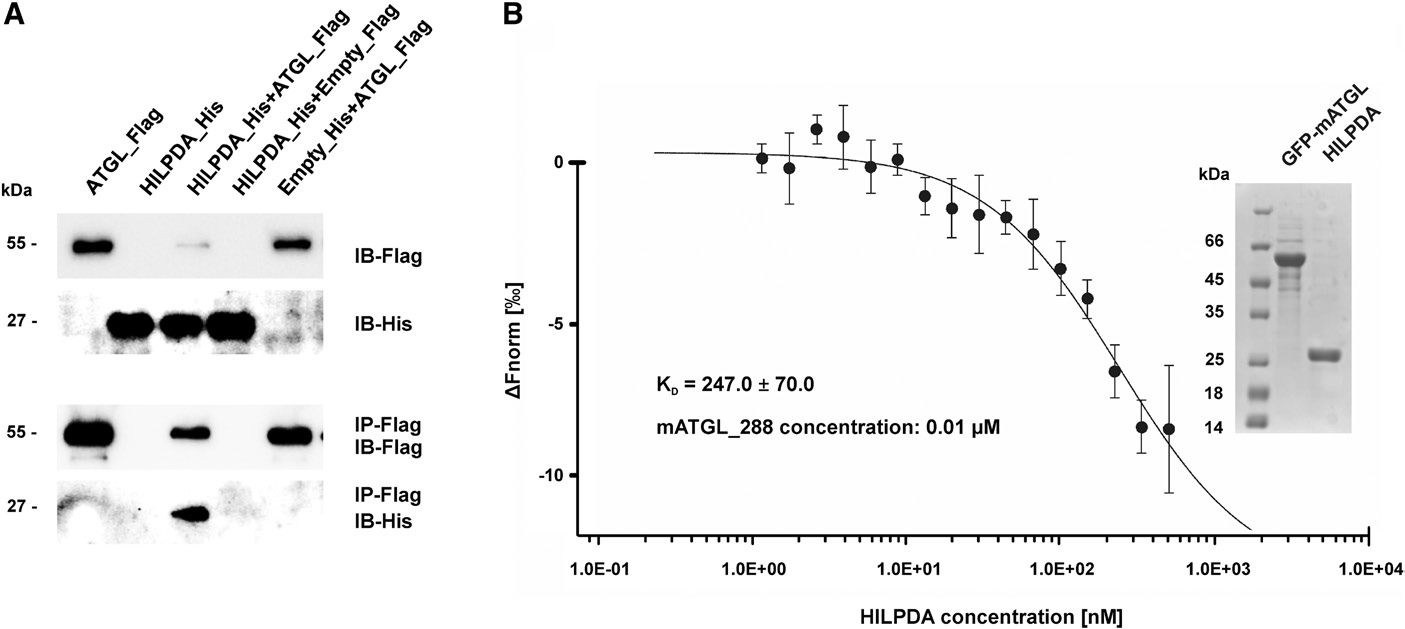
HILPDA directly interacts with the N-terminal region of ATGL. A: Coimmunoprecipitation of mATGL_FL and HILPDA. HEK293 cells overexpressing HILPDA and mATGL separately or in combination were lysed and incubated with antibodies directed against the Flag epitope for immunoprecipitation. Equal amounts of cell protein or immunoprecipitates were analyzed for the presence of antigens by immunoblotting. B: ATGL/HILPDA interaction demonstrated by MST. 0.01 μmoles of purified sf-GFP_mATGL_288 was titrated against increasing amounts of unlabeled smt3_HILPDA_FL and fluorescence distribution changes over a temperature gradient were measured using the Monolith NT.115. A Coomassie stained SDS-PAGE gel of the purified proteins used in the MST measurement is shown in the inset.

To assess whether HILPDA inhibition of ATGL requires direct protein-protein interaction, we performed coimmunoprecipitation experiments with HEK293 cell lysates containing mATGL_FL_flag and GFP_hHILPDA_His variants. A monoclonal anti-FLAG antibody bound to Sepharose beads coimmunoprecipitated HILPDA along with ATGL (**Fig. 4A**). Conversely, no HILPDA was immunoprecipitated when expressed alone or with empty control constructs, arguing for a direct molecular interaction between ATGL and HILPDA.

To confirm this observation by an independent experimental approach, we investigated the HILPDA-ATGL interaction by MST with purified proteins (33). This technique depends on the directed motion of a molecule through a temperature gradient induced by an infrared laser. The monitored thermophoresis of a protein typically differs significantly from the thermophoresis of a protein-protein complex due to changes in size, charge, and solvation energy. The technique allows the analysis of a fluorescence label. In our experiments, green fluorescent protein was N-terminally fused to ATGL to allow for analysis by fluorescence. MST analysis revealed ATGL-HILPDA binding with a calculated K_D_ of 247 μM (Fig. 4B). No binding was observed when ATGL was titrated against smt3 protein alone (data not shown). The purified proteins used for MST analysis are shown in the inset.

### HILPDA and ATGL colocalize and physically interact intracellularly

**Fig. 5.**
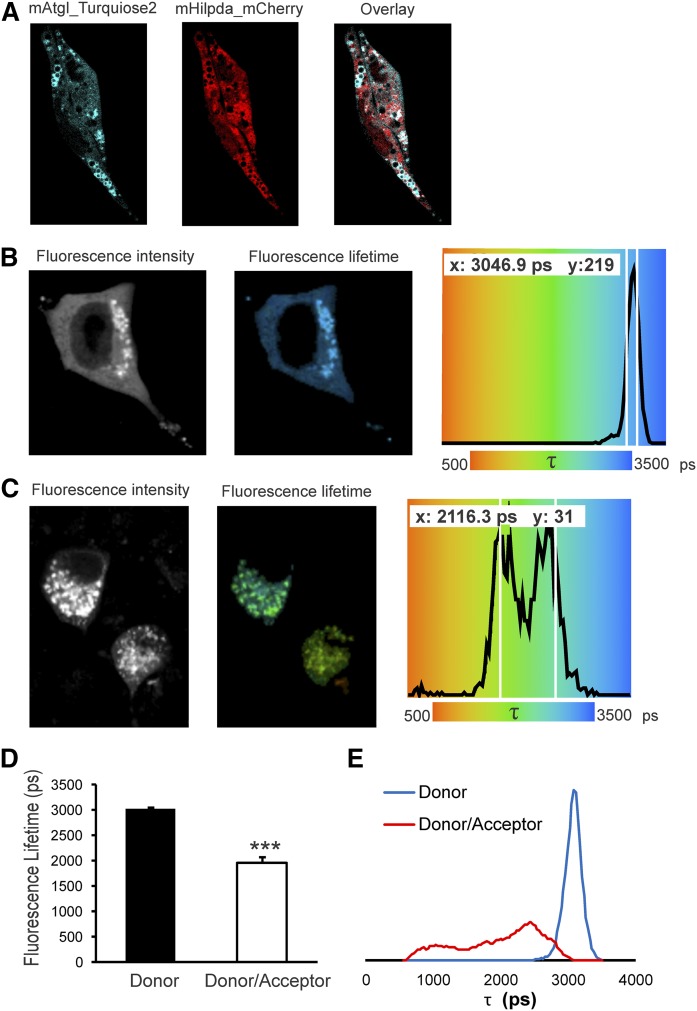
HILPDA and ATGL colocalize and physically interact intracellularly. Hepa 1-6 cells were transfected with mATGL-mTurquoise2 and mHILPDA-sYFP2 or mHILPDA-mCherry plasmids under lipid loaded conditions (1.2 mM fatty acid mix oleate and palmitate). Colocalization was observed by confocal microscopy and ATGL and HILPDA interaction was assessed using FRET-FLIM. A: mATGL_mTurquoise2 and mHILPDA_mCherry partially colocalize in lipid loaded Hepa 1-6 cells. B: Fluorescence intensity image, fluorescence lifetime image, and fluorescence lifetime distribution of mATGL-mTurquoise2 transfected cell. C: Fluorescence intensity image, fluorescence lifetime image, and fluorescence lifetime distribution of mATGL-mTurquoise2 and mHILPDA-sYFP2 cotransfected cell. D: Histogram of average fluorescence lifetime (τ) of mATGL_mTurquiose2 (N = 20) in absence and presence of acceptor mHILPDA_sYFP2 (N = 21). E: Average τ distribution histogram in absence (N = 20) and presence of acceptor (N = 21). Data are presented as mean ± SEM (***P < 0.001).

To further confirm the interaction between HILPDA and ATGL, we performed confocal microscopy and FRET-FLIM in Hepa 1-6 cells (**Fig. 5**). We transfected Hepa 1-6 cells with mHILPDA_mCherry and mATGL_mTurquoise2 and observed that HILPDA partially colocalized with ATGL (Fig. 5A). Because confocal microscopy is diffraction limited to ∼250 nm, our colocalization results did not directly prove that HILPDA and ATGL are physically interacting. To demonstrate protein interactions, we performed FRET quantified by FLIM. FRET is a process in which the excitation energy is transferred from a donor fluorophore to an acceptor chromophore through nonradiative dipole-dipole coupling when the fluorescent donor and acceptor molecules are in very close proximity (<10 nm). FRET determined using FLIM is independent of protein concentration, but very sensitive to the local microenvironment of the fluorophores. In FRET-FLIM, the fluorescence lifetime of the donor molecule is reduced in the presence of an acceptor molecule nearby, because energy transfer to the acceptor will introduce an additional relaxation path from the excited to the ground state of the donor (34). In our experiments, the donor fluorophore, mTurquoise2, was fused to ATGL and the acceptor, sYFP2, was fused to HILPDA. The donor fluorophore, mATGL_mTurquoise2, displayed a mean fluorescence lifetime (τ) of about 3,000 ps in the absence of acceptor (see Fig. 5B; left, fluorescence intensity; middle, corresponding false colored fluorescence lifetime image; right, distribution of fluorescence lifetimes). The donor alone showed a narrow distribution. However, when mATGL_mTurquoise2 was expressed in the presence of mHILPDA-sYFP2, a strong reduction (35%, *P* < 0.001) in donor fluorescence lifetime was observed (Fig. 5C, D). Interestingly, the average fluorescence lifetime distribution of the donor in the presence of acceptor showed a wider distribution (Fig. 5E), suggesting different populations of interacting species.

### HILPDA inhibits ATGL with an IC_50_ value in the low micromolar range

**Fig. 6.**
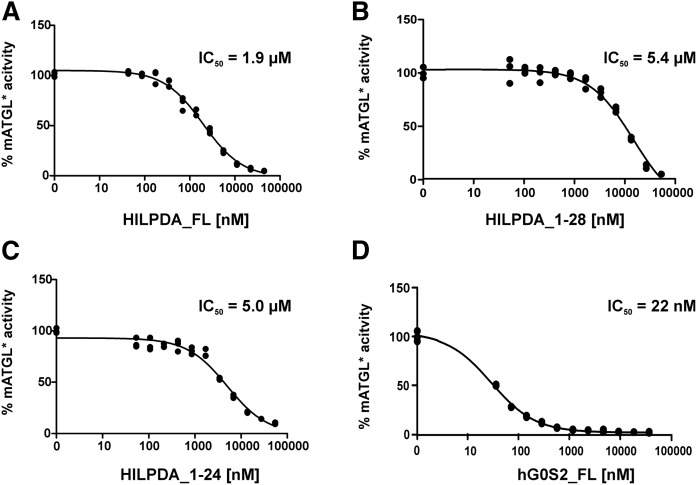
HILPDA inhibits ATGL with an IC_50_ value in the low micromolar range. Dose-dependent inhibition of TGH activity of mATGL288 by HILPDA variants and G0S2. 44 nM to 44 μM of Purified HILPDA (44 nM to 44 μM) or purified G0S2 (37 nM to 37 μM) was mixed with 25 μg mATGL288 lysate in the presence of CGI-58 and TG hydrolase assays were performed. Full-length HILPDA (A); HILPDA_1-28 (B); HILPDA_1-24 (C); and full-length hG0S2 (D).

To determine the IC_50_ of HILPDA, lysates of mATGL288-expressing cells were incubated with increasing concentrations of purified full-length HILPDA. As shown in **Fig. 6A**, hHILPDA inhibited mATGL in a concentration-dependent manner and an IC_50_ value of 1.9 μM. The C-terminal truncations, hHILPDA_1-28 (Fig. 6B) and hHILPDA_1-24 (Fig. 6C), exhibited similar inhibition capacities (IC_50_ values of 5.0 and 5.4 μM, respectively). The IC_50_ value for hG0S2 inhibition of ATGL is 22 nM (Fig. 6D) under the same experimental conditions and in agreement to previous reports (9). This approximately 100-fold difference suggests that G0S2 is a more potent inhibitor for ATGL than HILPDA.

### HILPDA is a highly selective inhibitor for ATGL

**Fig. 7.**
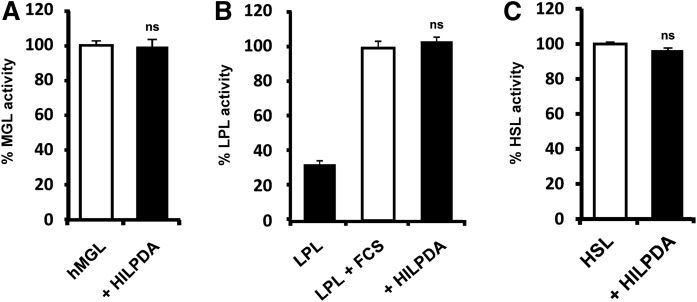
HILPDA selectively inhibits ATGL. MGL activity was measured using *rac*-(1,3)-monooleylglycerol as substrate. TGH activity of LPL and HSL was determined using triolein as substrate. All assays were performed in the absence or presence of purified full-length HILPDA. A: Forty nanograms of purified human MGL were mixed with 1.3 μg HILPDA (100% activity corresponds to 0.5 mmol glycerol/h*mg protein). B: Thirty-three nanograms of purified bovine LPL were stimulated with 10 μl of FCS and assayed in the absence or presence of 2.5 μg of HILPDA (100% activity corresponds to 1.1 mmol FA/h *mg protein). C: Twenty-five micrograms of HEK293 cell lysate expressing HSL-ECFP were incubated in the presence of 5 μg HILPDA (100% activity corresponds to 183 nmol FA/h *mg protein).

Truncation studies of HILPDA indicated that the predominantly hydrophobic N-terminal region of HILPDA is essential for ATGL inhibition. Because hydrophobic regions are prone for nonspecific protein-protein interactions, we analyzed the selectivity of HILPDA for the inhibition of different lipolytic enzymes. The screen included purified human MGL, purified bovine LPL, and lysates of mHSL expressing HEK293 cells. HILPDA did not inhibit any of these lipases in enzyme activity assays (**Fig. 7**). Hence, we conclude that HILPDA is a selective protein inhibitor of ATGL.

## DISCUSSION

Hypoxic conditions upregulate HILPDA expression and this response is often associated with increased TAG and LD accumulation. The data presented here clearly show that HILPDA can directly affect TAG catabolism by inhibition of ATGL. Decreased lipolytic activity can trigger metabolic effects in the organism, including decreased generation of lipotoxic intermediates and decreased rates of oxygen-demanding fatty acid oxidation. The inhibitory effect of HILPDA on ATGL and TAG catabolism has been the subject of controversy. It is conceivable that the herein demonstrated IC_50_ value of 2 μM of HILPDA for ATGL inhibition might explain the modest effects observed in literature reports. Similar IC_50_ values have been reported for ATGL inhibition with long-chain acyl-CoA (28). We also identified the N-terminal region harboring the first 20 amino acids of HILPDA as the mediator for ATGL inhibition. ATGL, the target of HILPDA inhibition, consists of approximately 500 amino acids. Previous studies have demonstrated that the N-terminal patatin domain-containing part of the protein is essential and sufficient for TAG hydrolysis, interaction with the coactivator protein, CGI-58/ABHD5, the murine-specific synthetic inhibitor, Atglistatin, acyl-CoA, and the inhibitory protein, G0S2 (9, 10, 28, 35). This study revealed that HILPDA also targets the patatin domain-containing region of ATGL. Inhibition of the TAG-hydrolyzing activity of ATGL by HILPDA occurs independently of the presence of CGI-58, suggesting that binding of both proteins to ATGL is not mutually exclusive. Direct protein-protein interaction between HILPDA and ATGL is supported by immunoprecipitation experiments with full-length ATGL, TAG-hydrolysis studies, MST experiments with ATGL truncated at residue 288, and FRET-FLIM analysis. We also showed that HILPDA is not a general inhibitor of lipases. While it inhibits ATGL activity, it has no effect on the other major lipolytic enzymes, HSL and MGL, or the vascular TAG lipase, LPL.

The inhibitory function of HILPDA toward ATGL, along with its expression regulation, protein size, and amino acid composition, shares striking parallels with the previously characterized ATGL inhibitor, G0S2 (Fig. 1). G0S2 evolved earlier during evolution, as indicated by orthologs in sharks, *Latimeria*, gars, and later developed vertebrates. In contrast, BLAST searches indicate that HILPDA is present only in two of the three groups of mammals, namely in eutherian mammals and marsupials (*Monodelphis domestica*). No HILPDA ortholog has been reported for egg-laying mammals (monotremes). Whether the gene coding for HILPDA evolved from a gene duplication event that was tolerated and probably provided a fitness advantage under certain environmental conditions, or if it appeared independently from G0S2 remains a matter of speculation. The human genes coding for HILPDA and G0S2 are located on different chromosomes (chromosome 7 and chromosome 1, respectively). The encoded proteins differ in size by 40 amino acids, which is, considering the size of HILPDA with only 63 residues, a quite substantial difference. Yet, we and other laboratories could show that the highly conserved N-terminal region of G0S2 includes the key residues involved in the interaction (3, 9). In a similar manner, we could identify that the N-terminal region of HILPDA, which has the highest similarity to G0S2, is also sufficient to inhibit ATGL.

Previous reports identified HILPDA and G0S2 as LD-associated proteins and the corresponding genes being targets of PPARs (13–16, 18). Biochemical characterization, knockdown, and overexpression studies have uniformly demonstrated that G0S2 functions to attenuate ATGL-mediated lipolysis (9, 36, 37). Up to now, the effect of HILPDA in TAG catabolism had remained controversial. Depletion of HILPDA was reported to lower hepatic lipid accumulation, reduce fat pad weight, and result in loss of LD formation in macrophages (13, 14, 16, 18, 38). Dijk et al. (15) observed only modest changes in adipogenesis and lipolysis upon manipulations of HILPDA expression in adipocytes (18). Our in vitro experiments presented here demonstrate that, in analogy to the function of G0S2, HILPDA is a direct inhibitor of ATGL. However, dose-response curves showed a much higher IC_50_ value for HILPDA than for G0S2 (2 μM vs. 22 nM for HILPDA and G0S2, respectively). This relatively low inhibitory capacity raises the question on the physiological role of HILPDA in the temporal and spatial regulation of ATGL-mediated lipolysis. Nevertheless, it should be kept in mind that the data presented here are from in vitro experiments and, thus, might not directly reflect the physiological relevance of HILPDA-mediated ATGL inhibition, because the affinities and local concentrations might vary in the presence of artificial substrates and LDs. Because HILPDA is strongly induced under hypoxic conditions, it is conceivable that it regulates lipolysis, particularly under hypoxic or anaerobic conditions as often observed in rapidly growing malignant tumors. Similarly, HILPDA may regulate lipolysis during metabolic reprogramming when cells have restricted oxygen supply to enable higher ratios of adenosine triphosphate production per molecule of oxygen consumed and to reduce production of reactive oxygen species (39–41). In cultured myocytes, G0S2 serves as an enhancer of ATP synthase, thus enhancing cellular tolerance upon hypoxia (11). Whether HILPDA shares even more functions with G0S2 and also directly interacts with mitochondrial ATP synthase or other protein interaction partners of G0S2, such as nucleolin (42) and/or Bcl-2 (43), is currently unknown and awaits further investigation.

### Note added in proof

While this paper was under review, Zhang et al. reported inhibition of lipolysis by hypoxia-inducible gene 2 (DOI: 10.7554/eLife.31132), which is fully in agreement with the observations reported here.
